# Harnessing redox proteomics to study metabolic regulation and stress response in lignin-fed *Rhodococci*

**DOI:** 10.1186/s13068-023-02424-x

**Published:** 2023-11-20

**Authors:** Xiaolu Li, Austin Gluth, Song Feng, Wei-Jun Qian, Bin Yang

**Affiliations:** 1 Bioproducts, Sciences, and Engineering Laboratory, Department of Biological Systems Engineering, Washington State University, Richland, WA 99354 USA; 2 Biological Sciences Division, Pacific Northwest National Laboratory, Richland, WA 99352 USA

**Keywords:** Lignin degradation, *Rhodococcus*, Redox biology, Proteomics, Metabolic regulation

## Abstract

**Background:**

*Rhodococci* are studied for their bacterial ligninolytic capabilities and proclivity to accumulate lipids. Lignin utilization is a resource intensive process requiring a variety of redox active enzymes and cofactors for degradation as well as defense against the resulting toxic byproducts and oxidative conditions. Studying enzyme expression and regulation between carbon sources will help decode the metabolic rewiring that stymies lignin to lipid conversion in these bacteria. Herein, a redox proteomics approach was applied to investigate a fundamental driver of carbon catabolism and lipid anabolism: redox balance.

**Results:**

A consortium of *Rhodococcus* strains was employed in this study given its higher capacity for lignin degradation compared to monocultures. This consortium was grown on glucose vs. lignin under nitrogen limitation to study the importance of redox balance as it relates to nutrient availability. A modified bottom–up proteomics workflow was harnessed to acquire a general relationship between protein abundance and protein redox states. Global proteomics results affirm differential expression of enzymes involved in sugar metabolism vs. those involved in lignin degradation and aromatics metabolism. As reported previously, several enzymes in the lipid biosynthetic pathways were downregulated, whereas many involved in β-oxidation were upregulated. Interestingly, proteins involved in oxidative stress response were also upregulated perhaps in response to lignin degradation and aromatics catabolism, which require oxygen and reactive oxygen species and generate toxic byproducts. Enzymes displaying little-to-no change in abundance but differences in redox state were observed in various pathways for carbon utilization (e.g., β‑ketoadipate pathway), lipid metabolism, as well as nitrogen metabolism (e.g., purine scavenging/synthesis), suggesting potential mechanisms of redox-dependent regulation of metabolism.

**Conclusions:**

Efficient lipid production requires a steady carbon and energy flux while balancing fundamental requirements for enzyme production and cell maintenance. For lignin, we theorize that this balance is difficult to establish due to resource expenditure for enzyme production and stress response. This is supported by significant changes to protein abundances and protein cysteine oxidation in various metabolic pathways and redox processes.

**Supplementary Information:**

The online version contains supplementary material available at 10.1186/s13068-023-02424-x.

## Background

Bacteria of the genus *Rhodococcus* are promising microbial chassis for synthesis of fuels and chemicals using low-cost biomass derived substrates. They are well-known for their ligninolytic capabilities and capacity to produce lipids, which are valuable platform chemicals [1–3]. Under stressful conditions such as nitrogen limitation, oleaginous *Rhodococci* such as *R. jostii* RHA1 and *R. opacus* PD630 accumulate triacylglycerides (TAG) using certain carbon sources. To date research into the fundamentals of bacterial TAG synthesis has focused on carbohydrate utilization and the metabolic rearrangements implicated in supplying metabolic precursors and NADPH for lipogenesis [4–7]. Questions pertaining to the feasibility of lipid production using lignin, aromatics, and non-sugar compounds (e.g., furfural) have persisted [8–10]. Compared to lipid production using lignin model compounds or carbohydrates, *Rhodococci* grown on lignin produce substantially less lipids [11–13]. Lignin utilization is a resource intensive process exemplifying a costly tradeoff between enzyme production and cell biomass accumulation to maintain a balance between supplies and energy required for catabolism (oxidation) and those for anabolism (reduction).

Lignin is a complex heterogeneous polymer comprised of various aromatic subunits linked together by C–O–C and C–C bonds. A broad repertoire of redox active and accessory enzymes are employed for lignin depolymerization and aromatics metabolism [14]. *Rhodococci* express various peroxidases and accessory oxidases to depolymerize lignin. For example, *R. jostii* RHA1 employs the well-characterized dye-decolorizing peroxidase (DypB) [15]. DypB is a versatile lignin peroxidase that requires peroxide for activity and directly uses phenolics and manganese ions as free radical mediators for lignin degradation. Following depolymerization, upper pathways funnel a wide variety of aromatics to the central aromatic intermediates catechol, protocatechuate, and gallate [16]. *R. opacus* PD630 and *R. jostii* RHA1 use β‑ketoadipate, phenylacetic acid, and other central pathways to aerobically cleave aromatics and ultimately produce central metabolites [9].

Co-cultivation of different microbial strains can enhance utilization of biomass-derived substrates for improved growth and bioproduct synthesis [17]. Compared to monocultures, a consortium of *Rhodococci* showed a higher capacity to degrade alkali lignin from corn stover potentially due to enzymatic synergism [9, 18]. Engineered *R. jostii* deficient in vanillate O-demethylase (VanA^−^) was employed to funnel lignin-derived aromatics to vanillate, which can hypothetically be used for lipid production by *R. opacus* [18–20]. Nevertheless, lignin to lipid yields for the consortium were comparable to those of monocultures [8]. Proteomics analysis was subsequently conducted to elucidate the molecular mechanisms conferring the emergent property of increased lignin degradation and to explore differences in the expressed metabolism of glucose-fed vs. lignin-fed cultures [9]. Pathways related to carbohydrate metabolism, including glycolysis, the pentose phosphate (PP) pathway, and the Entner–Doudoroff (ED) pathway, were greatly downregulated using lignin as the sole carbon source under nitrogen limitation. These pathways can provide NADPH, glycerol 3-phosphate, and acetyl-CoA for TAG synthesis [4, 21]. Fatty acid β-oxidation was likely upregulated to produce NADH and acetyl-CoA for growth as well as enzymes for lignin utilization. TAG synthesis enzymes were largely downregulated during lignin fermentation [9].

Reactive oxygen species (ROS) such as hydrogen peroxide (H_2_O_2_) are implicated in lignin and aromatics utilization [22, 23]. Interestingly, antioxidant enzymes are upregulated during lignin utilization: these include thioredoxin, catalase, and superoxide dismutase, which compete with fatty acid synthesis for NADPH [9, 24]. Lipid metabolism is intrinsically tied to the redox state of *Rhodococcus* [4, 25, 26]. Costa et al. reported a group of fatty acid synthesis proteins that were differentially oxidized at cysteine thiols [4]. Thiol redox post-translational modifications (PTM) can alter protein activities to regulate biological processes and/or protect against oxidative damage [27–30]. Redox PTMs generally occur as reversible oxidation of cysteine thiol groups and include S-mycothionylation (SSM), S-sulfenylation (SOH), disulfide bonds, etc. [25, 31]. The regulatory interplay between lignin catabolism, oxidative stress, and lipid metabolism is still uncharacterized. We hypothesize that redox-dependent mechanisms modulate carbon metabolism. To address this, a LC–MS/MS-based proteomics approach was applied to measure protein abundance and cysteine thiol oxidation (i.e., protein redox state) in the same experiment [32]. The redox proteomes of a *Rhodococcus* consortium were quantitatively compared for glucose vs. lignin growth conditions—providing the first, direct evidence of redox-dependent PTMs as a function of carbon source.

## Results

### Protein abundance patterns during cultivation on lignin vs. glucose

**Fig. 1:**
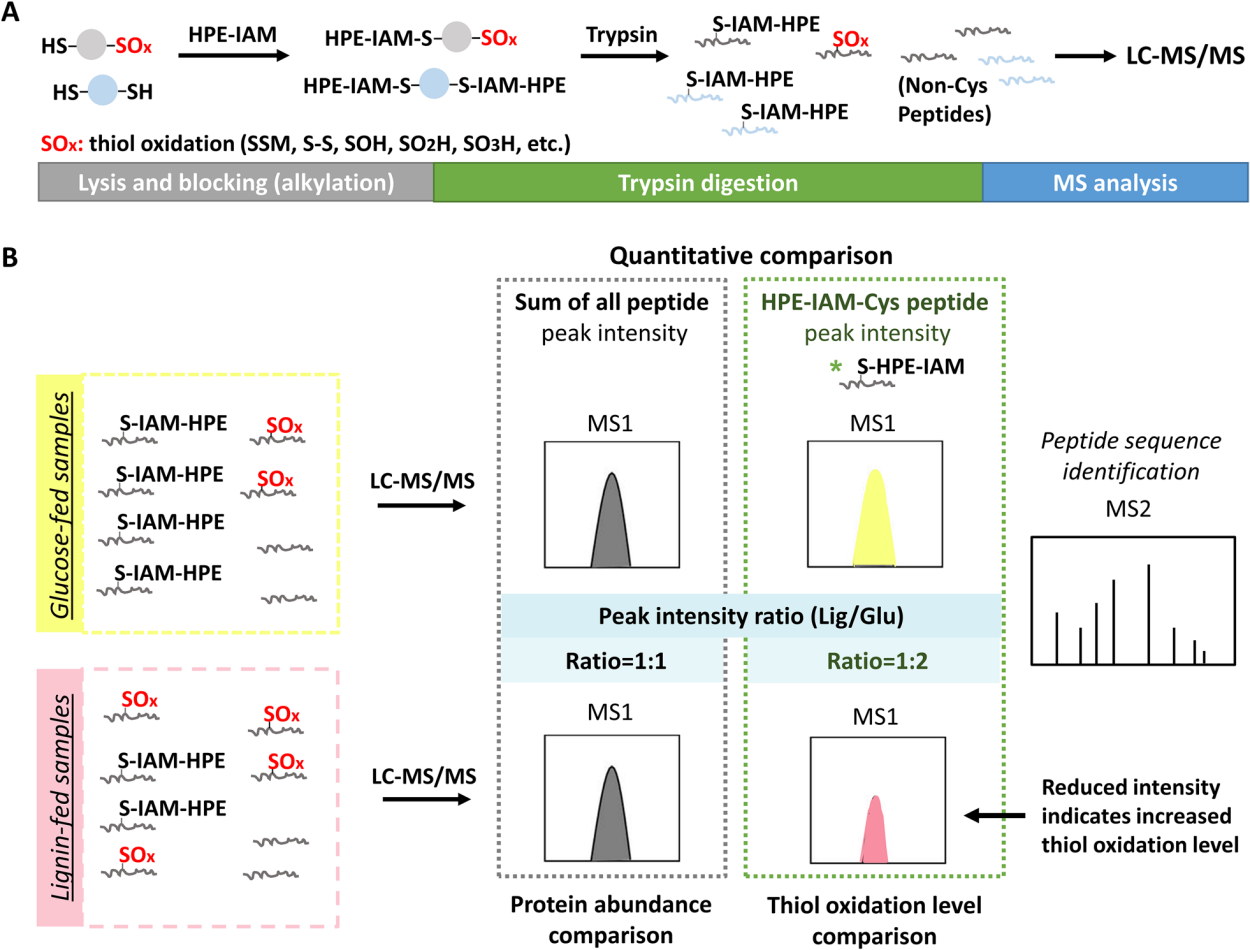
Quantification of protein abundance and cysteine thiol oxidation levels in *Rhodococci* fed on glucose or lignin as sole carbon sources. **A** Proteomics sample preparation. Proteins were extracted in the presence of HPE-IAM. Cysteine free thiols (SH) were alkylated with HPE-IAM while oxidized cysteine residues (e.g. SOH, SSM, S–S, SO_2_H, SO_3_H) were preserved. Then, proteins were digested for MS analysis. **B** Simultaneous relative quantification of protein abundance and thiol oxidation level. Peptide samples from glucose- or lignin-fed *Rhodococci* were subjected to MS analysis. The sum of MS1 peak intensities of all peptides assigned to individual proteins were used to compare a given protein’s abundance between two conditions. The peak intensities of HPE-IAM alkylated Cys-containing (HPE-IAM-Cys) peptides were summed for individual protein Cys residues, showing the abundance of protein cysteines at reduced state, which can be used to compare the thiol oxidation level of a given Cys site between two conditions. Note that the assay provides an indirect measurement of thiol oxidation level. The higher intensities of HPE-IAM-Cys peptides indicate lower cysteine thiol oxidation levels and vice versa

**Fig. 2:**
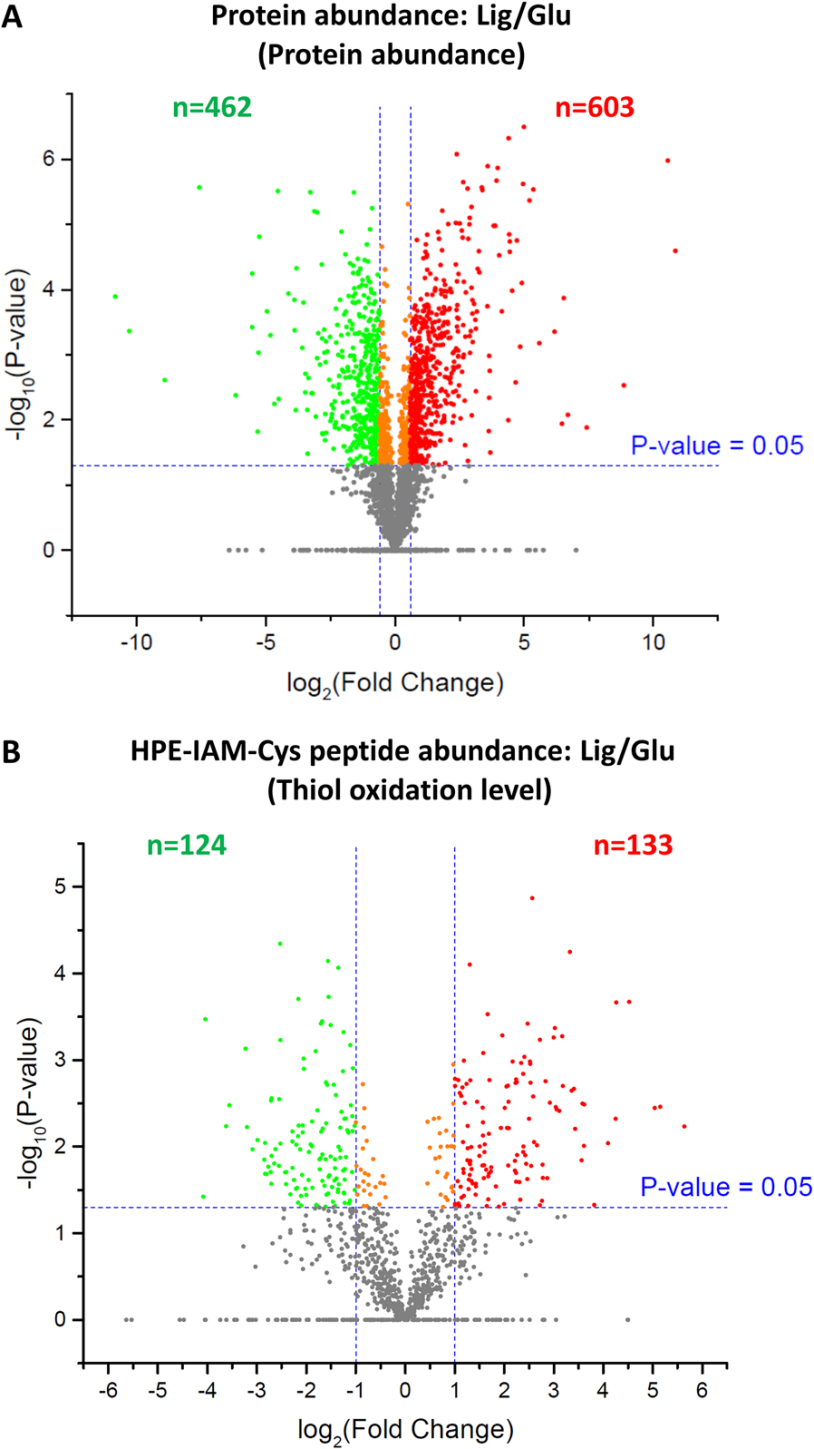
Relative quantification of protein abundance and HPE-IAM-Cys peptide abundance (i.e. cysteine thiol oxidation level). **A** Volcano plot comparing protein abundances in *Rhodococci* samples during lignin vs. glucose fermentations. Proteins with significantly changed abundances were indicated in red (upregulated) or green (downregulated). Criteria was applied: adjusted *p*-value < 0.05, fold-change > 1.5. **B** Volcano plot comparing cysteine thiol oxidation levels of individual proteins in *Rhodococci* samples for the aforementioned conditions. Protein cysteine sites with significantly altered oxidation levels were indicated in red (reduced oxidation) or green (increased oxidation). Criteria was applied: adjusted *p*-value < 0.05, fold-change > 1.5

A recently reported LC–MS/MS-based direct detection workflow was adapted for this study to simultaneously quantify protein abundances and protein cysteine thiol oxidation [32]. This is accomplished by omitting enrichment steps for cysteine-containing peptides. This analytical approach was used to study a *Rhodococcus* synthetic consortium (*R. jostii* RHA1, *R. opacus* PD630, and *R jostii* RHA1 VanA^–^) grown on 5 g/L glucose or lignin as the sole carbon source under nitrogen-limitation [9, 32]. Following cell lysis, reduced cysteine free thiols were blocked with the alkylation agent HPE-IAM to minimize oxidation during sample preparation (Fig. 1A). Oxidized thiol PTMs including disulfide (S–S), SSM, SO_2_H, and SO_3_H are comparably stable under mild conditions, mostly preserving them during sample preparation [27]. In contrast to the published direct detection method, which focused on exploring multiple types of cysteine PTMs, this study harnesses the MS intensities of HPE-IAM alkylated cysteine-containing peptides (HPE-IAM-Cys) to determine protein oxidation level. Proteins were considered as “having lower oxidation levels” when the corresponding HPE-IAM-Cys peptides were detected with higher MS intensities (Fig. 1B). In total 3682 proteins were identified using our LC–MS/MS workflow. A higher coverage of protein identification and quantification was achieved compared to the previous label-free proteomic analysis using filter-aided sample preparation [9]. Compared to the glucose condition, 603 proteins were upregulated and 462 proteins downregulated in the lignin condition (fold-change > 1.5, Student’s *t*-test *q*-value < 0.05) (Fig. 2A).

Consistent with our previous findings, enzymes involved in lignin depolymerization and upper aromatics pathways were observed (Additional file 1: Table S1). This includes the peroxidase DypB; however, significant differences in abundance were not observed likely because the secretomes were not analyzed [15]. Cytochrome P450 (CYP) was also observed but only in the lignin condition. This heme-thiolated monooxygenase is involved in demethylation and/or dealkylation of alkoxybenzoates such as guaiacol [33]. In *R. rhodochrous*, CYP is part of a two-component system with two redox partners, ferredoxin and ferredoxin reductase (upregulated up to 21.5-fold in our results) [34]. The products of this reaction are catechol and formaldehyde—the latter being an example of a toxic byproduct generated during lignin degradation. Enzymes in central aromatic degradation pathways including the β-ketoadipate pathway (both catechol and protocatechuate branches), phenylacetic acid pathway, and homogentisate pathway were significantly upregulated (up to 14.9-fold) in the lignin condition. Catechol 2,3-dioxygenase and 2-keto-4-pentenoate hydratase, which catalyze meta-cleavage of catechol, were upregulated.

Enzymes that produce and cycle reactive oxidants to attack lignin via Fenton chemistry were also upregulated in *Rhodococci* [35, 36]. These include glycolate oxidase, quinone reductases (up to 28.7 fold), NAD(P)H dehydrogenase, and cholesterol oxidase [37–42]. Glycolate oxidase is a flavin mononucleotide (FMN)-dependent enzyme that catabolizes phenylglyoxal and mandelic acid substrates as well as toxic glycolaldehyde byproducts [40]. Expression of these enzymes as well as the generation of toxic byproducts from lignin degradation may partially explain the upregulation of oxidative stress response proteins including catalases, alkyl hydroperoxide reductases, and a cold shock protein [25, 43]. Some proteins involved in the synthesis and degradation of mycothiol (MSH, a low-molecular-weight antioxidant) were also more abundant [44]. Corroborated by the results of Hensen et al., a MSH-dependent enzyme crucial for detoxifying formaldehyde, a byproduct of guaiacol and vanillin catabolism, was upregulated 3.8 fold. This dehydrogenase produces S-formylmycothiol and NADPH [23]. These results suggest competing NADPH requirements between lignin utilization and lipogenesis. In accordance with lignin depolymerization, proteins involved in central aromatic degradation pathways including the β-ketoadipate pathway (both catechol and protocatechuate branches), phenylacetic acid pathway, and homogentisate pathway were significantly upregulated (up to 14.9-fold) in the lignin condition. Catechol 2,3-dioxygenase and 2-keto-4-pentenoate hydratase, which catalyze meta-cleavage of catechol, were upregulated.

In addition to their structural role, lipids are secondary metabolites crucial for redox homeostasis and energy balance [26]. Plausibly induced by redox imbalance, enzymes involved in lipid metabolism were differentially expressed [25]. A number of proteins involved in β-oxidation were significantly upregulated (e.g., acetyl-CoA C-acyltransferase, upregulated up to 72.5 fold) during lignin conversion. A few proteins involved in fatty acid synthesis were upregulated in the lignin condition: these include FabG, a 3-oxoacyl-[acyl-carrier-protein] reductase; FabD, an [acyl-carrier-protein] S-malonyltransferase, and FabF, a 3-oxoacyl-[acyl-carrier-protein] synthase. These enzymes are components of the type II Fatty Acid Synthase (FAS-II), which elongates acyl-CoA to produce mycolic acids [45, 46]. Mycolic acids are characteristic constituents of Mycobacterial cell walls and modulate cell surface properties in response to the environment and stressors—including aromatics [47]. Several acyltransferases of the Kennedy pathway were downregulated, which supports the negligible lipid accumulation observed during lignin utilization. Glyceroneogenesis enzymes including glycerol-3-phosphate dehydrogenase were also downregulated.

### Differences in protein cysteine oxidation according to carbon source

**Fig. 3:**
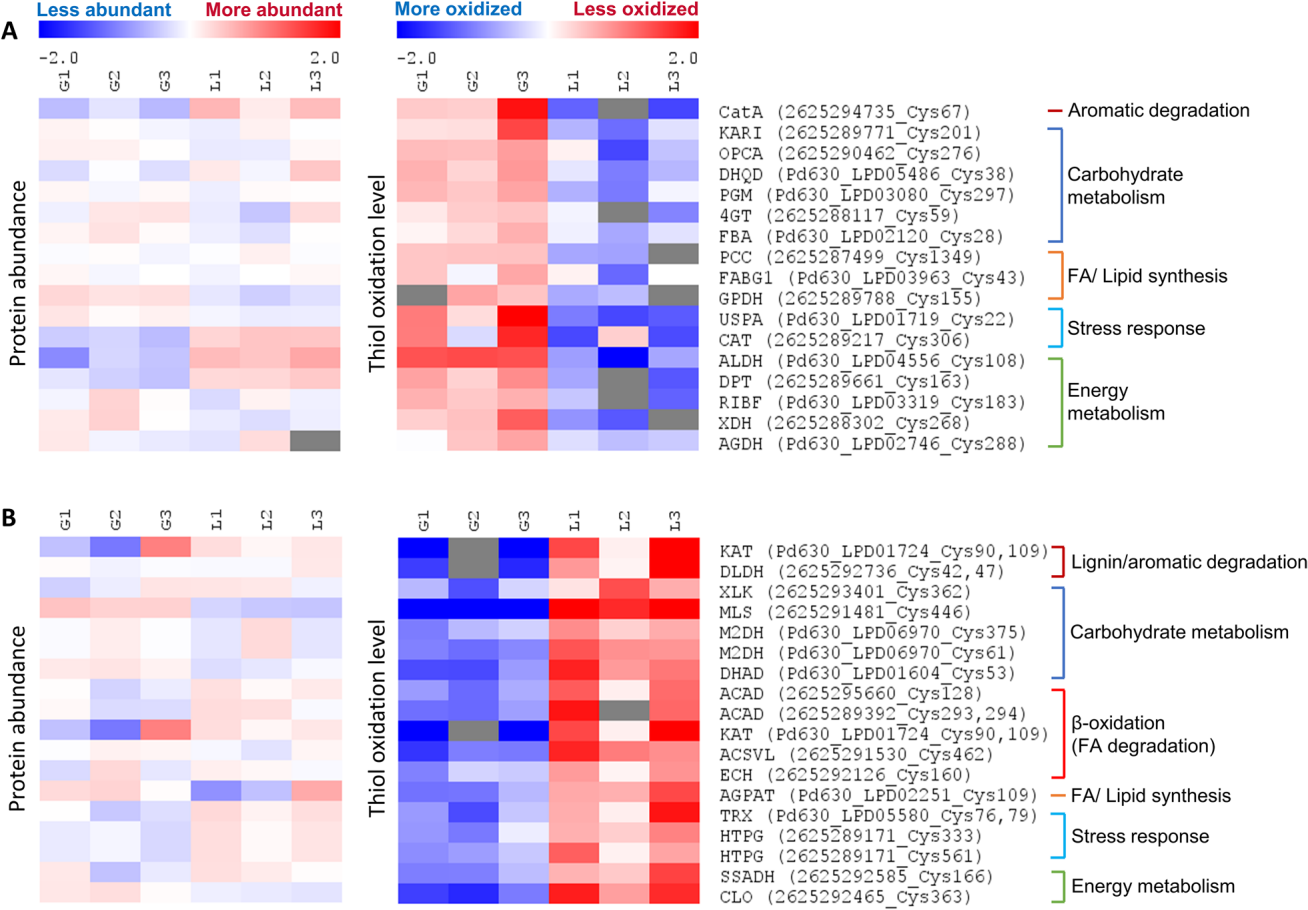
Overview of the differentially oxidized protein at cysteine site level (*p*-value < 0.05, and fold-change ≥ 1.5) among the lysate samples from glucose or lignin fermentation after 5 days. The ID prefixes correspond to the following: “Pd630” = *R. opacus* PD630 and “26,252…” = *R. jostii* RHA1. Left panel: protein abundance level; right panel: protein cysteine thiol oxidation level. Relative abundances (intensities) of proteins or HPE-IAM-Cys peptides were log2 transformed and median centered to zero. Each row represents one protein Cys site and each column represented one sample. “G1”, “G2” and “G3” are the lysate triplicate samples from glucose fermentation; “L1”, “L2” and “L3” are the lysate triplicate samples from lignin fermentation. All the fermentation was conducted by co-culture of three strains: *R. jostii* RHA1, *R. jostii* RHA1 vanA^−^, *R. opacus* PD630. The protein name abbreviation was followed by FASTA IDs of strains and Cys site IDs. **A** The selected proteins more oxidized during lignin fermentation. **B** The selected proteins more reduced during lignin fermentation

To evaluate protein redox states, we utilized an indirect approach whereby alkylated peptides would indicate original levels of reduced cysteine free thiols. Proteins assigned with higher intensities of HPE-IAM-Cys peptides (i.e., reduced Cys-containing peptides) were considered as having lower oxidation levels. Selection criteria were applied for both statistical significance (Student’s *t*-test *q*-value < 0.05) and fold change (at least 1.5-fold changes to HPE-IAM-Cys peptide intensities) (Fig. 2B). In total, 1668 alkylated cysteine-containing peptides were quantitatively compared. 133 HPE-IAM-Cys peptides showed higher abundance (i.e., lower oxidation levels) while 124 cysteine sites had higher oxidation levels for lignin vs. glucose fermentations. Some cysteine residues were represented by several HPE-IAM-Cys peptides, which requires additional data processing to faithfully represent the redox state for a given residue. Thus, protein redox states were further analyzed at the Cys site level by annotation and aggregation of the raw intensities of HPE-IAM-Cys peptides. The summed intensities of individual protein Cys sites were compared by Student’s t-test. Protein Cys sites with significantly changed oxidation level were filtered by: fold-change > 1.5, Student’s *t*-test raw *p*-value < 0.05. To differentiate redox state changes from protein abundance, only proteins with insignificant differences in abundance (– 1.5 < protein abundance fold-change < 1.5) were considered as candidates regulated according to their redox state. 162 differently oxidized protein Cys sites passed our criteria. These proteins were mainly involved in carbohydrate metabolism, lignin/aromatic degradation, lipid metabolism, stress response, amino acid metabolism, and energy balance (Fig. 3).

**Fig. 4:**
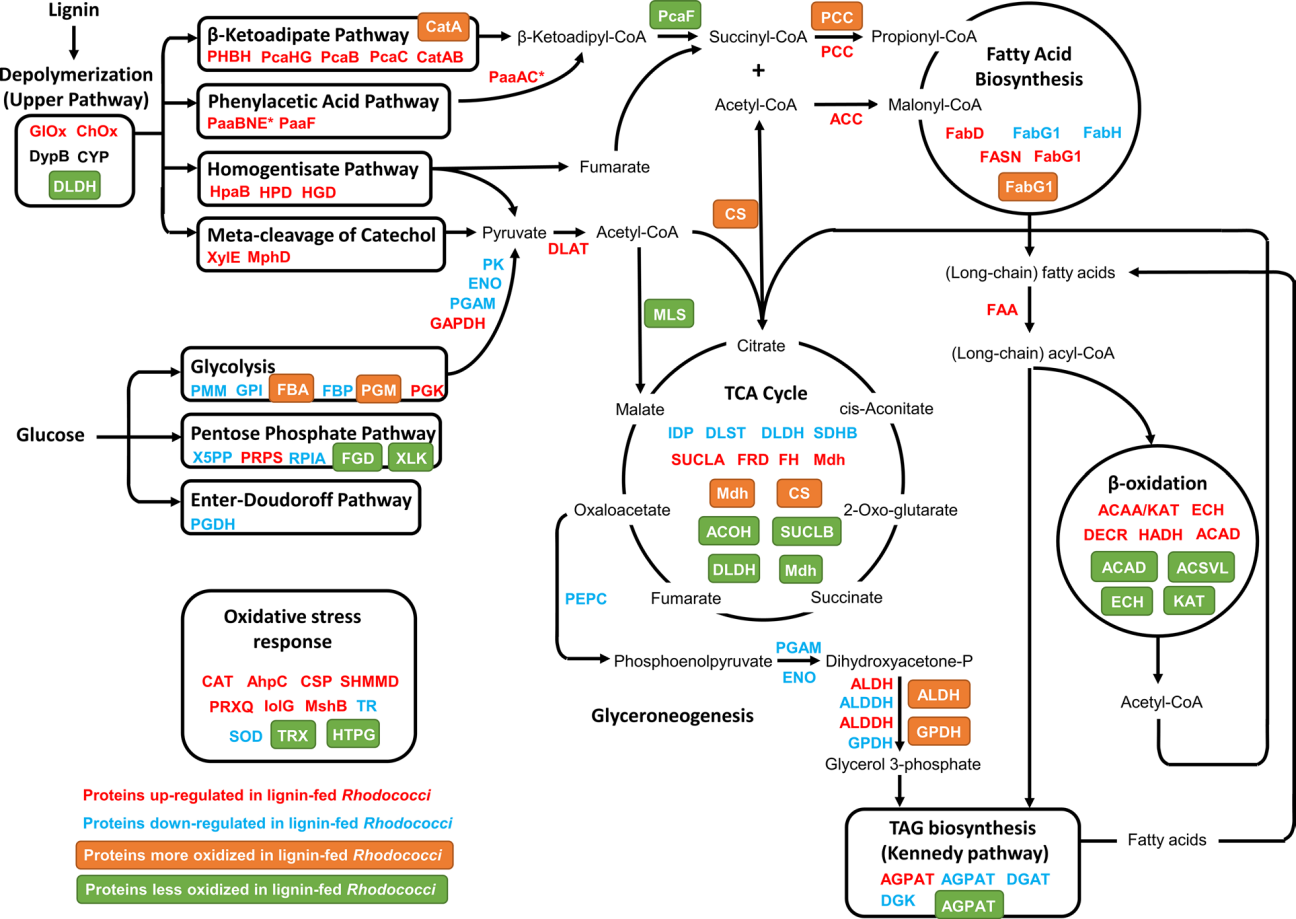
Overview of main metabolic network of lignin conversion to lipid in *Rhodococci*. Compared to samples from glucose fermentation, abbreviations for proteins upregulated in lignin-fed *Rhodococci* are presented in red (* indicates that the given protein was significantly upregulated in our previous [9] work), whereas downregulated proteins in lignin-fed *Rhodococci* are presented in blue; the abbreviations for proteins with increased oxidation levels at the reported cysteine thiols are shown in orange boxes, whereas protein cysteine sites with decreased oxidation level are shown in green boxes. Proteins written in black were observed but significant differences in expression were not

Without glucose or other sugars as carbon sources, a group of proteins involved in carbon metabolism (e.g., glycolysis and aromatics catbolism) were more oxidized during lignin fermentation: these include catechol 1,2-dioxygenase (CatA), fructose-bisphosphate aldolase (FBA), and phosphoglucomutase (PGM) (Fig. 3A). CatA is important for aromatics catabolism; it uses molecular oxygen and a non-heme reaction center for intradiol cleavage of catechol [48]. The oxidized Cys67 site is found in its conserved linker domain, which is involved in homodimerization according to protein sequence classification using InterPro and a structural analysis of a related species [49, 50]. It is possible that oxidized Cys67 affects the conformation of this domain and, as a result, phospholipid binding, dimerization, protein complex localization, and/or other functions; however, there are no other reports of this cysteine residue in literature. In yeast, FBA is partially oxidized during oxidative stress, thus affecting a variety of cellular pathways [51]. In *actinobacteria*, redundant FBA activity was observed suggesting a cycle between gluconeogenesis as well as the Entner–Doudoroff and pentose phosphate (PP) pathways [52, 53]. In contrast, malate synthase (MLS) was less oxidized, which may affect metabolic flux through the glyoxylate cycle, and thus the production of succinate and malate for gluconeogenesis (Fig. 3B). Pentose phosphate (PP) pathway enzymes F420-dependent glucose-6-phosphate dehydrogenase (FGD), and xylulokinase (XLK) were less oxidized (Figs. 3B and 4). Besides its obvious role in sugar metabolism, the PP pathway is crucial for coping with oxidative stress and provides intermediates for fatty acid synthesis [3]. FGD is reportedly involved in an F420-depedent anti-oxidant mechanism for bacterial stress response [54].

An enzyme central to energy metabolism, dihydrolipoamide dehydrogenase (DLDH), showed decreased oxidation levels for Cys42 and Cys47 during lignin conversion (6.37-fold change in intensity). DLDH is ubiquitous for its role as a subunit of the pyruvate dehydrogenase complex, α-keto glutarate dehydrogenase complex, and branched chain amino acid dehydrogenase complex—many of which require the antioxidant cofactor α-lipoic acid [55]. The DLDH catalytic mechanism involves NAD^+^ reduction and FADH_2_ oxidation cycles for cysteine disulfide bond formation [56, 57]. According to UniProt, Cys42 and Cys47 are within the active site and tend to form a redox-sensitive disulfide bond [58]. The activity of this protein is reversibly altered by H_2_O_2_ and reducing agents [59]. Recently, Rahmanpour et al. reported that DLDH in *Thermobifida fusca* prevented in vitro lignin re-polymerization [60]. The capture of reduced DLDH during lignin conversion suggests a multifaceted role in ROS scavenging, lignin degradation, and/or redox regulation of central metabolism.

The redox states of certain cysteine residues for enzymes involved in anabolism were also quantified. Two enzymes involved in fatty acid synthesis displayed increased oxidation in the lignin condition (Fig. 3A): acetyl/propionyl-CoA carboxylase alpha unit (ACC/PCC) and another component of FAS-II, a 3-oxoacyl-[acyl-carrier-protein] reductase (FabG1). ACC/PCC is involved in de novo fatty acid synthesis. In *S. cerevisiae*, this protein’s enzymatic activity can be attenuated in a redox-controlled fashion [51]. In *E. coli*, redox-sensitive components of FAS-II (e.g., FabF) were oxidized during nitrosative stress [61]. Interestingly, both these proteins are less oxidized during nitrogen-limitation (supporting TAG accumulation) compared to nitrogen abundance (limiting TAG accumulation) in *R. jostii* RHA1 [4]. In this study, *Rhodococci* were grown in nitrogen-limited conditions; nevertheless, using lignin as the sole carbon source still led to a significant shift towards oxidized fatty acid synthesis enzymes. Ultimately, a reduced state may be required for carbon flux to lipids.

Two acyl-CoA dehydrogenases (ACAD) were less oxidized in lignin-fed *Rhodococci* samples. ACAD catalyzes the first step in each cycle of β-oxidation to break down fatty acids. In eukaryotes, there is evidence suggesting that the activity of this enzyme is decreased due to cysteine PTMs (e.g., oxidation, alkylation, etc.): it is possible that this regulatory mechanism is conserved in prokaryotes [62, 63]. Furthermore, a 3-ketoacyl-CoA thiolase (ACAA/KAT), which catalyzes the thiolytic cleavage of 3-ketoacyl-CoA into acyl-CoA and acetyl-CoA during β-oxidation, was also less oxidized (18.5-fold higher intensity of HPE-IAM-Cys peptides) during lignin fermentation. Notably, ACAA/KAT also catalyzes the last step of β-ketoadipate pathway converting β-ketoadipyl-CoA to acetyl-CoA and succinyl-CoA. ACAA/KAT redox-regulation in plants and bacteria involves reversible formation of a disulfide bond between two catalytic cysteines [64, 65]. Under conditions conducive to oxidation, disulfide bond formation leads to a conformational change and inactivation. Active site residues for *Rhodococcus* ACAA/KAT were predicted using NCBI and UniProt sequence alignments and yielded Cys109, His401, and Cys431 [58, 66]. In our results, Cys109 was less oxidized (a free thiol instead of a disulfide bond), suggesting higher activity for fatty acid and aromatic degradation during lignin conversion.

In addition to carbon assimilation and lipid metabolism, differential oxidation of enzymes involved in amino acid and purine metabolism were observed. Amino acid and purine scavenging pathways generate energy and metabolic precursors for regeneration/synthesis of NAD and various other molecules. These pathways also provide endogenous sources of nitrogen during nitrogen limitation [2–4]. NADP^+^-dependent succinate-semialdehyde dehydrogenase (SSADH), which is involved in glutamate degradation, catalyzes the conversion of succinate-semialdehyde to succinate and regenerates NADPH as a result. Decreased oxidation of SSADH was observed in the lignin condition hinting at a redox regulatory mechanism sensitive to the available carbon source under nitrogen limitation. This is also supported by the lower oxidation state of adenosine deaminase, involved in purine scavenging, and a putative enamine deaminase (RidA), which gets rid of reactive enamine intermediates [67, 68]. These intermediates are generated by pyridoxal 5′-phosphate-dependent enzymes such as ornithine aminotransferase and phosphoserine aminotransferase (both less oxidized in our results). Interestingly, xanthine dehydrogenase, which converts xanthine to urate, can be converted to the ROS-generating oxidase form via reversible oxidation [69]. A final example includes the first enzyme in the shikimate pathway, 3-deoxy-D-arabinoheptulosonate 7-phosphate synthase, which exhibited 9.01-fold lower oxidation. This pathway is important for metabolism of aromatic amino acids such as tryptophan which is required for de novo NAD synthesis [70]. These results showcase the complexity by which protein activity is regulated to modulate availability of essential nitrogenous metabolites (e.g., cofactors and amino acids).

Besides metabolic enyzmes, several transcriptional regulators were differentially oxidized, two of which belong to the two-component systems (TCS). This suggests redox regulation of TCS components which impact signal transduction and metabolism at the transcriptional level in response to environment changes such as nitrogen limitation and carbon source availability [71, 72]. The TCS response regulator GlnR is a global regulator with a central role in nitrogen metabolism. This regulator has been reported in other *Mycobacteria* [73, 74]. The transcriptional regulator NnaR, which can be activated by GlnR, is a co-activator associated with nitrate/nitrite assimilation. NnaR orthologues have been found in *R. jostii* and *R. opacus* and are named NlpR. NlpR exhibits functionality in modulating lipogenesis and lipid accumulation in addition to ammonium limitation [5]. This implies an important role of the GlnR-mediated system in lipid accumulation for oleaginous *Rhodococci*. Another TCS consisting of histidine kinase PrrB and response regulator PrrA was reported in *Mycobacterium smegmatis* and regulates expression of several genes involved in TAG and lipid biosynthesis pathways [75]. Unfortunately, little is known about redox regulation of TCS and its partners.

Redox regulation has been proposed for stress conditions such as nitrogen-limitation and even in the absence of stress [30]. It is well established that thioredoxin (Trx) and glutathione-glutaredoxin antioxidant systems mediate redox homeostasis in eukaryotes [76, 77]. The exposed active 2-Cys sites of these proteins reduce oxidized proteins via thiol-disulfide exchange reactions. Similar mechanisms were proposed for the MSH-mycoredoxin (Mrx) system in Gram-positive bacteria [44]. In our results, several proteins involved in MSH synthesis and metabolism were upregulated during lignin fermentation. Furthermore, peroxiredoxin and alkyl hydroperoxide reductases (AhpC), which are important scavengers of H_2_O_2_ and peroxide-functionalized molecules, were upregulated during lignin fermentation [78]. Although protein abundances of the Trx system remained unchanged or downregulated, a putative thioredoxin 2-Cys site (Cys76 and Cys79) indicated decreased oxidation level in lignin-fed samples suggesting an active antioxidant defense (Fig. 3B). Meanwhile, a chaperone protein HtpG (a bacterial homolog of the eukaryotic chaperone Hsp90 which is involved in response to many environmental stresses) also showed decreased oxidation (up to 2.63 folds) at two Cys sites [79]. The role of these antioxidants in redox regulation of *Rhodococcus* metabolism requires further investigation.

## Discussion

Our preliminary results present a pattern of putative redox-dependent protein regulation that modulates a variety of metabolic pathways and biological processes (Fig. 4). Ultimately, differences in protein redox states track well with changes in abundance for corresponding biological processes. Firstly, a number of proteins in aromatic degradation pathways increased in abundance while PcaF and MLS were less oxidized, supporting catabolism of aromatics for TCA anaplerosis. Secondly, in addition to higher oxidation of PCC and FabG, downregulation of FAS and other fatty acid synthesis enzymes hints at a reduced anabolic flux from central metabolites to lipogenesis. Thirdly, proteins involved in β-oxidation and acetyl-CoA conversion (i.e., MLS) showed higher abundance and lower oxidation (separately, given the aforementioned filter criteria) evincing increased fatty acid degradation to maintain flux to the TCA cycle. Lastly, most glyceroneogenesis and Kennedy pathway proteins showed lower abundance pointing to decreased TAG synthesis. The orchestration of these carbon metabolism modules and those detailed for nitrogen metabolism (e.g., purine scavenging/synthesis) supports a regime for generating and cycling central metabolites and energy to build and maintain cell biomass instead of accumulating lipids. This metabolic redistribution seems correlated with oxidative stress response, but a causal link was not determined. Further investigations of oxidative stress and metabolism using lignin will be required to probe these relationships.

Profiling redox PTMs is a powerful first step towards investigating their potential regulatory roles. Future research will harness molecular approaches to specify antioxidant-enzyme interactions, redox switches, and the functional consequences of redox states. Immunoprecipitation is a widely used approach to identify protein–protein interactions: this mature technology may be used to co-precipitate antioxidants and their binding partners [80, 81]. Even though limited information is available for the identified proteins, bioinformatics and modeling tools can be used to predict cysteine site exposure, which affects their reactivity [31]. Direct mutagenesis and activity assays can be used to study individual proteins of importance to elucidate functional changes caused by redox PTMs and interrogate hypothetical redox switches for metabolic regulation. Promoting reducing power generation or enhancing antioxidant activities during lignin conversion may also improve lipid yields in *Rhodococci—*especially for demanding carbon sources like aromatics and lignin [82]. One novel approach for regenerating reducing power could be supplementing *Rhococcus* cultures with hydrogen (perhaps from a hydrogen-producing microorganism). Our results confirm expression and differential oxidation of a cytoplasmic [NiFe(Se)]-hydrogenase [83–85].

Our study of co-cultured *Rhodococci* provides intriguing metabolic insights and a platform for discovering candidate proteins involved in redox regulatory networks. Moreover, this study exemplifies how proteomics can be used to study synthetic microbial consortia, even though sequence similarity among the strains employed herein makes this challenging [86]. In future work, we plan to explore and validate select Cys site modifications using the open search strategy with FragPipe and targeted redox proteomics methods [87, 88]. Furthermore, we endeavor to qualify mechanisms of microbial interactions using metabolomics [17, 89].

## Conclusions

Efficient bacterial lipid production requires a steady carbon and energy flux to generate acetyl-CoA, glycerol-3-phosphate, and NADPH, while balancing fundamental requirements for enzyme production and cell maintenance. For lignin, we theorize that this balance is difficult to establish due to resource expenditure for enzyme production and oxidative stress response, the latter of which competes for NADPH. To study redox state as a function of carbon source, we investigated the expressed metabolisms of a synthetic *Rhodococcus* consortium grown on alkali lignin vs. glucose under nitrogen-limited conditions. A novel mass spectrometry-based detection workflow allowed us to pinpoint putative redox regulatory nodes in metabolic pathways by simultaneously quantifying protein abundances and redox states. Independent of abundance, several proteins in both conditions were differentially oxidized providing possible targets for further study. Additional studies of ROS, oxidants like lipid peroxides, and the MSH/MSSM ratio will further our understanding of redox imbalance and regulation during lignin utilization. Functional studies using targeted mutagenesis, molecular cloning, and activity assays will be required to confirm redox regulation of the reported proteins and tease out contributions to redox imbalance from lignin utilization vs. nitrogen starvation. This study exemplifies a unique perspective of microbial metabolism one can attain using redox proteomics: specifically, that PTMs are implicated in the tug-and-pull of oxidation and reduction, which lie at the heart of metabolism.

## Methods

### Alkali lignin preparation

Alkali-extracted lignin from corn stover was prepared as previously described [9, 18]. Briefly, lignin-rich solids containing 20% glucan, 11% xylan, 3% arabinan, 2% galactan, 53% lignin, and 11% ash were first obtained by treating corn stover with 0.1 M NaOH at 80 °C for 2 h. Then, lignin was solubilized by soaking lignin-rich solids in 0.1 M NaOH at pH 12.5 again. The supernatant was filtered filtered through 11 μm pore size Whatman filters. Lignin was recovered from the filtrate by slowly adjusting the pH to 3 with 2 M H_2_SO_4_. Precipitated lignin was collected and washed twice with 70 °C deionized water by filtration, then lyophilized for 3 days. Cellulose and hemicellulose fractions were not observed in the final alkali-extracted lignin [18]. The alkali lignin consisted of aromatic p-hydroxyphenyl (H), guaiacyl (G) and syringyl (S) units, and major lignin linkages (β-O-4, β-β, and β-5) as reported in the our previous work [18].

### *Rhodococci* cultivation

Co-cultivation of three *Rhodococcus* strains (*R. opacus* PD630, *R. jostii* RHA1, and its mutant *R. jostii* RHA1 VanA-) was conducted as previously described.^1^ Briefly, seed cultures for each strain were inoculated at 5% (v/v) into M9 medium with supplements and incubated at 30 °C, 180 rpm for 5 days. 5 g/L of glucose or alkali corn stover lignin was used as sole carbon sources. Ammonium sulfate was added as a nitrogen source at a C/N ratio = 15/1 (g/g). After fermentation, cells were pelleted by centrifugation, washed twice with NaCl solution (0.9%, w/v), and then processed for LC–MS/MS.

### Proteomics sample preparation

After fermentation, cells were pelleted by centrifugation at 8000 × *g* and 4 °C for 15 min, then washed twice with NaCl solution (0.9%, w/v). Cell pellets were resuspended in 10% (w/v) trichloroacetic acid (TCA) followed by incubation on ice for 20 min to partially lyse cells and preserve the redox proteome [90]. Precipitated proteins and cell debris were pelleted by centrifugation at 13,000 *g* for 15 min at 4 °C. The pellet was washed with 500 μl of ice-cold 10% TCA and then with 200 μl ice-cold 5% TCA. Then the pellet was resuspended in lysis buffer (250 mM HEPES, 10 mM EDTA, 0.5% SDS, 8 M urea, 10 mM HPE-IAM, pH 7.5) by intermittent sonication and incubation at 37 °C for 2 h [32, 90]. Bead-beating was performed using 100 μl of 0.1-mm zirconia/silica beads to further lyse cells and extract proteins. Cell lysate was centrifuged at 14,000 *g* for 10 min at 4 °C to remove cellular debris. The supernatant was incubated at 37 °C for 30 min for complete alkylation followed by acetone precipitation. The resultant protein pellet was dissolved in 25 mM ammonium bicarbonate buffer containing 8 M urea (pH 8) then subjected to FASP Protein Digestion Kit for lignin removal and trypsin digestion. All samples were cleaned up by C18 SPE column and concentrated by a Speed Vac SC110 following the manufacturer’s instructions. Samples were reconstituted to 0.1 µg/µL with 0.1% formic acid for LC–MS/MS analysis.

### LC–MS/MS analysis

Three biological replicates of samples were analyzed by a nanoAcquity ultra performance liquid chromatography (UPLC) system (Waters) coupled to a Q-Exactive HF Mass Spectrometer (Thermo Scientific, San Jose, CA) as previously described [91]. Protein identification and label-free quantification (LFQ) was conducted using MaxLFQ algorithm offered by MaxQuant [92], searching against FASTA files (*R. opacus* PD630, Accession: PRJNA30413; *R. jostii* RHA1, Accession: PRJNA309609) from NCBI and JGI databases [93, 94]. Dynamic oxidation of methionine (15.9949 Da) and dynamic HPE-IAM modification of Cys (177.0790 Da) were used for searching.

### Data analysis

LFQ intensities of proteins exported from MaxQuant were log2 transformed and compared by Student’s *t*-test values adjusted for Permutation-based false discovery rate in Perseus [95]. Significant protein abundance changes met the following criteria: (a) Student’s *t*-test *q*-value < 0.05; (b) fold-change > 1.5 or < –1.5. Protein redox state (i.e., oxidation states of cysteine thiols) was compared at peptide level by quantification of HPE-IAM-Cys peptides. Raw intensities of unoxidized cysteine-containing peptides (with add-on mass of HPE-IAM moiety) exported from MaxQuant were log2 transformed and normalized by median-center normalization across conditions, followed by Student’s *t*-test in Perseus. Protein redox state was quantified at Cys site level by annotation of Cys site of individual HPE-IAM-Cys peptides and aggregation of raw intensities of peptides with the same Cys sites. Then, Cys site intensities were log2 transformed and normalized, followed by student’s *t*-test by R. Protein cysteines with significantly increased or reduced oxidation level must pass the following criteria: (a) Student’s *t*-test raw *p*-value < 0.05; (b) fold-change of Cys site intensities > 1.5 or < –1.5; (c) log2 (fold-change) of corresponding protein abundance > –1.5 and < 1.5.

### Supplementary Information

**Additional file 1: Table S1.** Global protein abundances. **Table S2.** Proteins with significantly changed abundance. **Table S3.** Quantification of unique HPE-IAM alkylated cysteine sites. **Table S4.** Cys sites with significantly changed oxidation level.

## Data Availability

All data generated or analyzed during this study are included in this published article and its supplementary information files.

## Abbreviations

4GT: 4-Alpha-glucanotransferase
ABCT: Carbohydrate ABC transporter ATP-binding protein, CUT1 family
ACAD: Acyl-CoA dehydrogenase
ACOH: Aconitate hydratase
ACSVL: Putative (very) long chain acyl-CoA synthase
AGDH: Putative arogenate/prephenate dehydrogenase
AGPAT: 1-Acyl-sn-glycerol-3-phosphate acyltransferase
AhpC: Alkyl hydroperoxide reductase subunit C
ALDDH: Aldehyde dehydrogenase
ALDH: NAD-dependent alcohol dehydrogenase
ASADH: Aspartate-semialdehyde dehydrogenase
CAT: Catalase
CatA: Catechol 1,2-dioxygenase
CatB: Muconate cycloisomerase
CLO: Choline oxidase
CS: Citrate synthase
CSP: Cold-shock DNA-binding protein family
DGAT: Diacylglycerol O-acyltransferase
DGK: Diacylglycerol kinase
DHAD: Dihydroxy-acid dehydratase 1
DHQD: 3-Dehydroquinate dehydratase
DLAT: Pyruvate dehydrogenase E2 component (dihydrolipoamide acetyltransferase)
DLDH: Dihydrolipoamide dehydrogenase
DLST: 2-Oxoglutarate dehydrogenase E2 component (dihydrolipoamide succinyltransferase)
DPT: NAD^+^ diphosphatase
ECH: Enoyl-CoA hydratase
ENO: Enolase
FAA: Long-chain-fatty-acid–CoA ligase/fatty-acyl-CoA synthase
FabD: [Acyl-carrier-protein] S-malonyltransferase
FABG1: 3-Oxoacyl-[acyl-carrier-protein] reductase FabG1
FabH: 3-Oxoacyl-[acyl-carrier-protein] synthase 3
FASN: Fatty acid synthase
FBA: Fructose-bisphosphate aldolase
FBP: Fructose-1,6-bisphosphatase II
FGD: F420-dependent glucose-6-phosphate dehydrogenase
FH: Fumarate hydratase class I, aerobic;fumarase, class I, homodimeric
FRD: Fumarate reductase iron-sulfur subunit/fumarate reductase membrane anchor subunit/Fumarate reductase flavoprotein subunit
GAPDH: Glyceraldehyde-3-phosphate dehydrogenase
GK: Glucokinase
GPDH: Glycerol-3-phosphate dehydrogenase
GPI: Glucose-6-phosphate isomerase
HADH: 3-Hydroxyacyl-CoA dehydrogenase
HGD: Homogentisate 1,2-dioxygenase
HpaB: 4-Hydroxyphenylacetate 3-monooxygenase oxygenase component
HPD: 4-Hydroxyphenylpyruvate dioxygenase
HTPG: Molecular chaperone HtpG
IDP: Isocitrate dehydrogenase
IMPD: IMP dehydrogenase
IolG: Myo-inositol 2-dehydrogenase/ D-chiro-inositol 1-dehydrogenase, iolG
KARI: Ketol-acid reductoisomerase
KAT/ACAA/PcaF: 3-Ketoacyl-CoA thiolase/ Acetyl-CoA acetyltransferase/ β-Ketoadipate:succinyl-CoA thiolase, PcaF
M2DH: Mannitol 2-dehydrogenase
Mdh: Malate dehydrogenase (quinone)
MhpD: 2-Keto-4-pentenoate hydratase/2-oxohepta-3-ene-1,7-dioic acid hydratase
MLS: Malate synthase
MshB: 1D-myo-inositol 2-acetamido-2-deoxy-alpha-D-glucopyranoside deacetylase
OPCA: Glucose-6-phosphate dehydrogenase assembly protein OpcA
PaaAC: Ring 1,2-phenylacetyl-CoA epoxidase
PaaBNE: Phenylacetate-CoA oxygenase
PaaF: Phenylacetate-CoA ligase
PcaC: 4-Carboxymuconolactone decarboxylase/ 3-oxoadipate enol-lactonase
PcaHG: Protocatechuate 3,4-dioxygenase
PCC: Acetyl/propionyl-CoA carboxylase, alpha subunit
PEPC: Phosphoenolpyruvate carboxykinase
PGAM: Phosphoglycerate mutase (2,3-diphosphoglycerate-dependent)
PGDH: 6-Phosphogluconate dehydrogenase
PGK: Polyphosphate glucokinase
PGM: Phosphoglucomutase
PHBH: 4-Hydroxybenzoate 3-monooxygenase
PK: Pyruvate kinase
PMM: Phosphomannomutase
PRPS: Ribose-phosphate pyrophosphokinase
PRXQ: Peroxiredoxin Q/BCP
RIBF: Riboflavin biosynthesis protein ribF
RPIA: Ribose-5-phosphate isomerase
SDHB: Succinate dehydrogenase subunit B
SHMMD: S-(Hydroxymethyl)mycothiol dehydrogenase
SOD: Superoxide dismutase
SSADH: Succinate-semialdehyde dehydrogenase
SUCLA: Succinyl-CoA ligase [ADP-forming] subunit alpha
SUCLB: Succinyl-CoA ligase [ADP-forming] subunit beta
TKTL: Transketolase
TR: Thioredoxin reductase
TRX: Putative thioredoxin
USPA: Nucleotide-binding universal stress protein, UspA family
X5PP: Xylulose-5-phosphate
XDH: CO or xanthine dehydrogenase, Mo-binding subunit
XLK: Xylulokinase
XylE: Catechol 2,3-dioxygenase
